# Deep Learning for Joint Adaptations of Transmission Rate and Payload Length in Vehicular Networks

**DOI:** 10.3390/s19051113

**Published:** 2019-03-05

**Authors:** Mohamed Elwekeil, Taotao Wang, Shengli Zhang

**Affiliations:** 1 College of Information Engineering, Shenzhen University, Shenzhen 518060, China; mohamed.elwekeil@szu.edu.cn or mohamed.elwekeil@el-eng.menofia.edu.eg (M.E.); zsl@szu.edu.cn (S.Z.); 2 Department of Electronics and Electrical Communications Engineering, Faculty of Electronic Engineering, Menoufia University, Menouf 32952, Egypt

**Keywords:** IEEE 802.11p, vehicular networks, deep learning, intelligent transportation system, frame length, adaptive modulation and coding

## Abstract

Recently, vehicular networks have emerged to facilitate intelligent transportation systems (ITS). They enable vehicles to communicate with each other in order to provide various services such as traffic safety, autonomous driving, and entertainments. The vehicle-to-vehicle (V2V) communication channel is doubly selective, where the channel changes within the transmission bandwidth and the frame duration. This necessitates robust algorithms to provide reliable V2V communications. In this paper, we propose a scheme that provides joint adaptive modulation, coding and payload length selection (AMCPLS) for V2V communications. Our AMCPLS scheme selects both the modulation and coding scheme (MCS) and the payload length of transmission frames for V2V communication links, according to the V2V channel condition. Our aim is to achieve both reliability and spectrum efficiency. Our proposed AMCPLS scheme improves the V2V effective throughput performance while satisfying a predefined frame error rate (FER). Furthermore, we present a deep learning approach that exploits deep convolutional neural networks (DCNN) for implementing the proposed AMCPLS. Simulation results reveal that the proposed DCNN-based AMCPLS approach outperforms other competing machine learning algorithms such as k-nearest neighbors (k-NN) and support vector machines (SVM) in terms of FER, effective throughput, and prediction time.

## 1. Introduction

Vehicular networks enable various applications such as road safety, traffic jam reporting, public aid units guiding, entertainments, and autonomous driving, etc. [1,2]. Vehicular networks aim to provide sustainable and reliable communications for smart vehicles. They are basic building blocks in the cooperative intelligent transportation systems (ITS) of future smart cities. The vehicle-to-vehicle (V2V) communication in vehicular networks was standardized in the form of an amendment to the IEEE 802.11 standard. Specifically, the IEEE 802.11p defines V2V augmentations to the IEEE 802.11 standard in order to enable dedicated short-range communication (DSRC) for vehicular networks [3].

In wireless communication systems, the varying nature of wireless channels calls for link adaptations in order to improve both the spectrum efficiency and the communication reliability. There are many link adaptation schemes developed using traditional model-based approaches (e.g., [4,5]). However, modeling practical wireless communication systems incorporating varying wireless channels is complicated. Therefore, these model-based approaches make approximations on the model and consequently the system performance will be impacted. Another possible approach is to establish look-up tables for link adaptations. However, in practical wireless systems with high dimensions, getting a look up table that cover the whole range of link quality metrics will be prohibitively huge. Recently, learning-based approaches are employed to improve the effectiveness of wireless link adaptations. The authors in [6] proposed a supervised learning approach for adaptive modulation and coding (AMC) in the IEEE 802.11n wireless local area networks (WLANs). The target is to select the best modulation and coding scheme (MCS) in order to maximize the throughput under certain frame error rate (FER) constraint. They have utilized the k-nearest neighbors (k-NN) classification algorithm for AMC. In [7], the authors solved the same AMC problem using the algorithm of support vector machines (SVM). However, the works in [6,7] both assumed nonrealistic assumptions such as fixed frame length, perfect synchronization, and perfect channel knowledge. The work in [8] presented a deep convolutional neural network (DCNN) approach for AMC in MIMO-OFDM systems. The authors considered practical timing synchronization, carrier frequency offset and channel estimation at receivers. However, the authors did not consider the effect of frame length. Furthermore, they evaluated their approach for indoor WLANs applications.

It should be noted that the V2V communications are more challenging compared to the indoor WLANs. The V2V communication channel is doubly selective (i.e., in both of the frequency and the time domains). This means that the channel fluctuates considerably within the transmission bandwidth (frequency-selective fading) and within the frame duration (fast fading) [9]. This is due to the combined effects of the multipath nature of V2V propagation channels, along with the high mobility of radio terminals in the vehicles and some scatterers around [10]. Thus, advanced link adaptation schemes are crucial for V2V communications in order to deal with the varying V2V links under the stringent V2V communication requirements, such as high effective throughput and reliability. For instance, an adaptive frame length scheme for vehicular networks has been presented in [11]. The authors in [11] show that the adaptive frame length scheme outperforms non-adaptive frame length schemes for vehicular networks in terms of throughput, retransmissions, and overheads. However, the authors in [11] did not consider the effect of the MCS.

Another challenging task for V2V communications is the channel estimation. In traditional channel estimation techniques, transmitters add training sequences, i.e., preamble, at the beginning of each frame; then, receivers can utilize this preamble to estimate the channel and use the channel estimates to decode the following data symbols in the same frame. However, this is not optimal for a quickly varying V2V channel, where the channel estimates from the preamble at the beginning of the frame may be significantly different from the actual channels encountered by the subsequent symbols within the same frame. As a consequence, utilizing the initial channel estimates from the preamble to decode the subsequent symbols in the frame may lead to decoding errors. This leads to a significant degradation in the frame error rate performance of V2V links. Therefore, channel tracking techniques have been presented such as [12,13], where the initial channel estimates from the training sequences of the preamble are updated within the duration of the frame. In particular, the work in [12] presents a number of dynamic data-aided equalization techniques. The authors in [12] evaluated these techniques by empirical experiments in practical V2V environments and concluded that their equalization techniques outperform traditional preamble-based least squares (LS) [14] and pilot aided equalizers. Furthermore, they reported that their spectral temporal averaging (STA) technique gives the best performance compared to other equalization techniques presented in their paper. However, the work in [12] considered only the MCS of the lowest data rate, namely $MCS_{0}$, where the binary phase shift keying (BPSK) modulation and $\frac{1}{2}$ channel coding rate were assumed. Furthermore, they did not consider the effect of the frame length. In [13], the authors extended the scheme of [12] to different MCSs of higher data rates and showed that the STA channel tracking algorithm outperforms the traditional preamble-based LS channel estimation. Moreover, they have studied the effect of different fixed payload lengths with different fixed MCSs on the V2V links. However, they did not study the effect of link adaptation schemes in either the MCS or the payload length on the performance of V2V communications.

Recently, the application of machine learning to vehicular networks has attracted the attention of the research community [15,16]. In this paper, we present a joint adaptive MCS and payload length selection (AMCPLS) scheme. The proposed AMCPLS attempts to select the payload length, the modulation order, and the channel coding rate based on the V2V channel condition so as to achieve both reliability and high effective throughput. While the concept of the AMCPLS can be applied to both the 5G-V2V and the DSRC IEEE 802.11p V2V, in this paper, we will concentrate on the DSRC IEEE 802.11p V2V since its standard is more well-established and its specifications are more well-defined [17,18]. Furthermore, we are concentrating on safety-related applications and it has been reported in the literature that the DSRC IEEE 802.11p V2V outperforms the 4G-LTE V2V for the safety-related applications [19,20]. The contribution of this paper can be summarized as follows.

- First, we develop a novel link adaptation scheme for V2V communications, namely the AMCPLS scheme, that enables transmitters to adaptively select not only the MCS but also the payload length of the transmission frame. We consider practical receivers that involve realistic timing synchronization, carrier frequency offset correction and channel estimation. The proposed AMCPLS scheme is evaluated under traditional preamble-based LS channel estimation [14] and STA channel tracking [13]. In both cases, our adaptive AMCPLS scheme outperforms the non-adaptive schemes in terms of FER and effective throughput.
- Moreover, we propose a machine learning framework for the proposed AMCPLS scheme, where past observations of channel state information (CSI) along with achieved FER and effective throughput are used as training data. This training data can be utilized to predict the best combination of the payload length, the modulation order, and the channel coding rate for future V2V transmissions by leveraging machine learning tools. Specifically, a deep convolutional neural network (DCNN) has been designed for implementing the proposed AMCPLS scheme. Our DCNN-based AMCPLS scheme can achieve better performance compared to other machine learning based schemes, (such as k-NN and SVM).

We performed extensive numerical evaluations to verify our DCNN-based AMCPLS scheme. The remainder of this paper is organized as follows. Section 2 presents an overview of the IEEE 802.11p standard and the considered system model. In Section 3, we discuss the joint adaptive MCS and payload length scheme for V2V communications. Then, Section 4 presents our deep learning approach for AMCPLS. After that, simulation results are shown in Section 5. Finally, Section 6 concludes the paper.

## 2. IEEE 802.11p Physical Layer Overview and System Model

In this section, we first provide the background about the IEEE 802.11p standard for DSRC in vehicular networks. Then, we present the system model.

### 2.1. IEEE 802.11p Physical Layer

The IEEE 802.11p operates on the 5.9 GHz frequency band and utilizes the orthogonal frequency division multiplexing (OFDM) [3]. It offers eight different MCSs ( i.e., the different combinations of quadrature amplitude modulation (QAM) order and channel coding rate) with data rates that range from 3 Mbps to 27 Mbps as summarized in Table 1 [13]. The bandwidth of the communication channel is 10 MHz. The OFDM symbol duration is 8 µs with inverse fast Fourier transform (IFFT) period of 6.4 µs and guard interval of 1.6 µs. The total number of OFDM subcarriers, *U*, is 64, out of them there are only $U_{D}=48$ data subcarriers and $U_{p}=4$ pilot subcarriers. The remaining $U_{N}=12$ subcarriers are null carriers. Here the number of the used subcarriers, $U_{s}=U_{D}+U_{p}$ is 52 [21].

**Figure 1:**
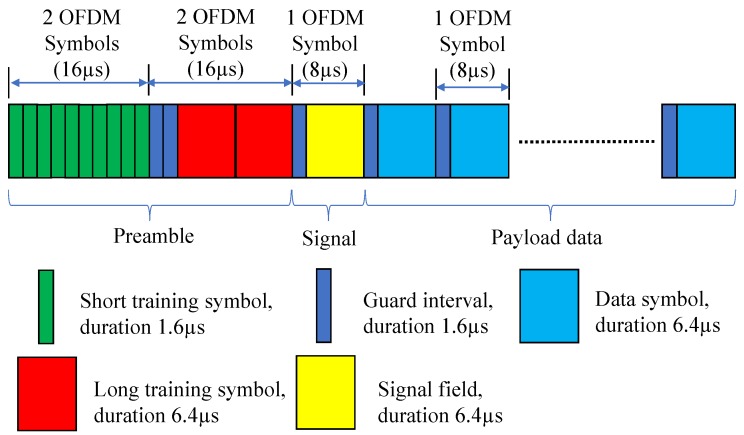
Frame structure of the IEEE 802.11p physical layer.

The IEEE 802.11p physical layer frame structure is shown in Figure 1. The frame starts with a preamble field that begins with 10 short training symbols (STSs), each of duration 1.6 µs. These STSs are used for timing synchronization and coarse frequency offset estimation. Then, the two long training symbols (LTSs), each has a duration of 6.4 µs, are added in order to be used by the receiver for fine frequency offset correction and channel estimation. After that, the signal field is used to add signaling information such as the utilized MCS (data rate) and the frame length. After the signal filed, the frame payload is added [13,21]. For a certain payload data of length *l* Bytes, the number of OFDM data symbols, $K_{D}$, in a frame can be given as [13]

(1) $$K_{D}=\left\lceil \frac{8l+b_{s}+b_{t}}{N_{DBOS}}\right\rceil ,$$

where $b_{s}$ is the number of service field bits, that equals 16 bits, added at the beginning of the payload data, $b_{t}$ denotes the number of tail bits, that is six bits, added at the end of the payload data [3], and $N_{DBOS}$ is the number of data bits per OFDM symbol for the utilized MCS as shown in Table 1. Here, operator $\left\lceil z\right\rceil$ is the ceiling function which returns the smallest integer that is larger than *z*. Note that there are five overhead OFDM symbols that carries no payload data are added in every frame, namely, the two OFDM symbols of the STSs, the two OFDM symbols of the LTSs, and one OFDM symbol of the signal field. Thus, the total number of OFDM symbols in a frame, *K*, is given by

(2) $$K=K_{D}+K_{ov},$$

where $K_{ov}$ is the number of overhead OFDM symbols per frame, that equals five for an IEEE 802.11p frame.

### 2.2. System Model

**Figure 2:**
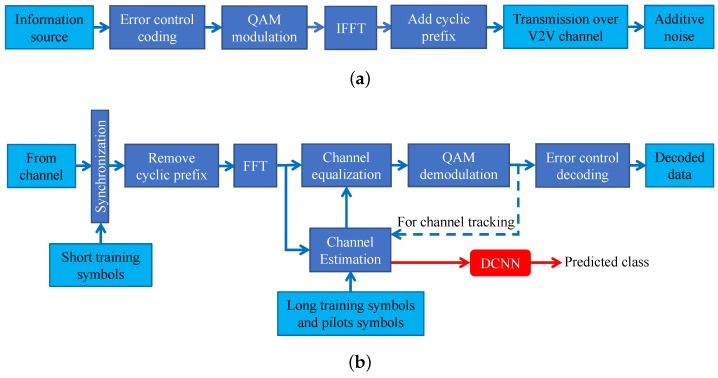
The block diagram of the IEEE 802.11p OFDM transmitter and receiver. (**a**) The IEEE 802.11p OFDM transmitter. (**b**) The proposed IEEE 802.11p OFDM receiver.

This part presents the system model of the IEEE 802.11p V2V transmitter and receiver. Specifically, Figure 2a shows the block diagram of the IEEE 802.11p transmitter. The transmitter first performs error control coding over the information bits using convolutional encoder with code rate, *c*. After that, the convolutionally encoded bits are modulated to the QAM symbols of order *M*. We use $x^{k}\left[n\right]\in C$ to denote the QAM modulated symbol transmitted on the subcarrier *n* of the OFDM symbol k∈{1,2,…,K}. Note that, the first five OFDM symbols (the preamble symbols and the signal field symbol), i.e., for k∈{1,2,3,4,5}, are overhead and do not carry any payload data. Then, the IFFT is performed on the QAM modulated symbols and the cyclic prefixes (CP) are added to generate the OFDM symbols. It should be noted that, at the transmitter side, pilots are inserted within every OFDM symbol in order to facilitate both carrier frequency recovery and channel tracking at the receiver. The OFDM symbols are transmitted over the doubly selective V2V channel. Here, we assume a frequency selective and fast fading channel, where the channel fading effect on the subcarrier *n* of the OFDM symbol *k* is described by the coefficient $H^{k}\left[n\right]\in C$.

The block diagram of the IEEE 802.11p receiver is shown in Figure 2b. The main operations implemented at the receiver are timing synchronization, frequency offset estimation, FFT, channel estimation, equalization, QAM demodulation and error control decoding. Particularly, the STSs are extracted from the frame preamble to perform frame timing synchronization and coarse frequency offset estimation and correction. Then, the CP of each OFDM symbol is removed and the FFT is preformed. After that, the frequency domain received symbol on the subcarrier *n* of the OFDM symbol *k* is denoted by $y^{k}\left[n\right]\in C$ that is given by

(3) $$y^{k}\left[n\right]=H^{k}\left[n\right]x^{k}\left[n\right]+v^{k}\left[n\right],$$

where $v^{k}\left[n\right]\in C$ is the complex additive white Gaussian noise (AWGN) with zero mean and $\sigma^{2}$ variance, i.e., $v^{k}\left[n\right]∼\mathrm{CN}\left(0,\sigma^{2}\right)$.

Then, the receiver performs the fine frequency offset estimation and correction using the LTSs. After that, utilizing the LTSs part of the frame preamble, the receiver obtains the estimated noise variance, ${\hat{\sigma }}^{2}$, and the estimated channel effect, ${\hat{H}}_{p}\left[n\right]$. The channel estimates ${\hat{H}}_{p}\left[n\right]$ are used for performing channel equalization on the received OFDM symbols $y^{k}\left[n\right]$. At the same time, the channel estimates ${\hat{H}}_{p}\left[n\right]$ for all subcarriers and the estimated noise standard deviation are used as the input features to a trained DCNN for predicting the suitable transmission mode class. Here, a transmission mode class refers to a combination of QAM modulation order, channel coding rate and data payload length. The predicted transmission mode class is sent to the transmitter to be considered for the next frame. After channel equalization, the QAM demodulation and error control decoding are executed to obtain the decoded data bits. In this paper, we consider two distinct channel equalization approaches. The first one is the traditional preamble-based channel equalization, where the channel estimates from the LTSs, ${\hat{H}}_{p}\left[n\right]$, are used for equalizing all OFDM symbols in the frame. The second approach is the STA channel tracking [13] where the channel estimates are continuously updated for every OFDM symbol in the frame so as to alleviate the effect of outdated channel estimates used for equalizing the subsequent OFDM data symbols.

The features of the current frame are extracted from the channel estimates ${\hat{H}}_{p}\left[n\right]$ and noise standard deviation $\hat{\sigma }$. These features are used as input to a pre-trained DCNN in order to predict the combination of QAM modulation order, channel coding rate and data payload length, that should be sent as feedback to the transmitter for using them in the forthcoming frame.

## 3. Joint MCS and Payload Length Adaptation for IEEE 802.11p V2V Networks

In this section, we present our joint adaptive modulation and coding scheme (MCS) and payload length selection (AMCPLS) for IEEE 802.11p V2V networks, where the communication channel is highly varying. Based on the channel condition, our AMCPLS approach selects both the MCS and the payload length. The aim of AMCPLS is to maximize the effective physical layer throughput and to fulfill a predefined frame error rate (FER).

We consider that the set of all available MCSs, I, is given by I={0,1,…,I−1}, where I=#I is the cardinality of I, which is the number of the available MCSs. The set of the allowable payload lengths, L, is given by $L=\{l_{0},l_{1},\ldots ,l_{L-1}\}$, where L=#L is the number of the allowable payload lengths. Assume that the MCS i∈I and the payload length l∈L are utilized. The total number of information data bits in a single IEEE 802.11p frame, $N_{DBF}$, is given by

(4) $$N_{DBF}=K_{D}\times N_{DBOS},$$

where $K_{D}$ is obtained from (1) and $N_{DBOS}$ is as shown in Table 1. Furthermore, the total IEEE 802.11p frame duration, $T_{f}$, is given by

(5) $$T_{f}=K\times T_{OS},$$

where $T_{OS}$ is the OFDM symbol duration which is 8 µs, and *K* is calculated as in (2). Thus, the effective data rate, $R_{eff}$, is given by

(6) $$R_{eff}=\frac{N_{DBF}}{T_{f}}=\frac{K_{D}N_{DBOS}}{\left(K_{D}+K_{ov}\right)T_{OS}},$$

Therefore, the effective physical layer throughput, Γ, is given by

(7) $$\Gamma =R_{eff}\left(1-FER\right)=\frac{K_{D}N_{DBOS}\left(1-FER\right)}{\left(K_{D}+K_{ov}\right)T_{OS}},$$

where FER is the frame error rate. Note that both $K_{ov}$ and $T_{OS}$ are constants for the IEEE 802.11p, and thus Γ does not depend on them. From Equation (7), it is clear that the three parameters that affect the effective physical layer throughput are $K_{D}$, $N_{DBOS}$, and FER. From (1), it is clear that $K_{D}$ is dependent on *l* and $N_{DBOS}$. Taking into account that $N_{DBOS}$ depends on the used MCS as detailed in Table 1, we can conclude that the effective physical layer throughput is dependent on the utilized MCS, the utilized payload length and the FER. Moreover, the FER is affected by three main factors, namely, the MCS, the payload length, and the transmission channel condition. Therefore, the effective physical layer throughput can be controlled by the MCS, the payload length, and the channel condition. The channel condition is determined by the surrounding wireless environment. This motivates us to jointly adapt both the MCS and the payload length based on the channel condition to maximize the effective physical layer throughput under a predefined FER constraint. The proposed AMCPLS scheme can be formulated as follows

(8) $$\left\{i^{*},l^{*}\right\}=\arg\max_{\left\{i,l\right\}}\left\{\Gamma \left(\left\{i,l\right\},g\right):FER\left(\left\{i,l\right\},g\right)<\epsilon \right\},$$

where ϵ is the target FER, g is the vector that represents the channel condition, and $\left\{i^{*},l^{*}\right\}$ is the optimum combination of the MCS and the payload length. The channel condition vector g needs to include the effects of both the channel fading and the AWGN.

We refer to the optimum solution of the problem expressed in (8) as the ideal AMCPLS. Practically, this ideal AMCPLS can not be implemented due to the following two obstacles. The first is that it is very challenging to correctly derive the FER of a wireless communication system, since the FER is a complicated function of the channel condition, the modulation and coding scheme, and the payload length. The second obstacle is that the actual channel condition, g, for the next frame cannot be assumed to be perfectly known in practice, since the channel is varying over different frames. In Section 5.1, we evaluate the ideal AMCPLS using numerical enumerations, where we assume that the channel condition for the next frame can be obtained. Although this solution to the ideal AMCPLS may not be practical, we treat the performance of this ideal AMCPLS as a benchmark. In next section, we resort to a deep learning approach to solve the AMCPLS scheme, which is more practical.

## 4. Deep Learning for AMCPLS in IEEE 802.11p V2V Networks

In this section, we present our deep learning approach to tackle the AMCPLS problem in IEEE 802.11p V2V networks. Generally speaking, a machine learning algorithm can learn the input-output relationship of a certain system directly from the previous observations (training data) without any previous knowledge about the model of the underlying system. If the adopted machine learning algorithm is sufficiently powerful, it can accurately learn the model of the underlying system utilizing the training data. Deep learning with deep nerual networks [22] has emerged as a powerful machine learning tool and has achieved impressive performance in many fields such as computer vision [23], speech recognition [24], and many other fields. Recently, the wireless communication community pays a considerable attention to deep learning in order to harness its capabilities in designing future wireless communication systems. In this work, we present a deep convolutional neural network (DCNN) approach for implementing AMCPLS in V2V networks. We formulate the AMCPLS problem as a multiclass classification problem and solve it by training the DCNN under the framework of supervised learning. For the multiclass classification problem, a certain class, a∈A, defines a combination of a MCS *i* and a payload length *l*, where A={0,1,2,…,A−1} is the set of the possible classes and A=I×L is the number of classes. The features are extracted from the estimated channel state information and estimated noise standard deviation. The target of our approach is to increase the effective throughput and satisfy a predefined FER constraint.

### 4.1. Features Preparation

For the sake of training our DCNN model, we need to construct a training set that includes *M* training examples, ${\left\{f^{m},a^{m}\right\}}_{m=1}^{M}$, where $f^{m}$ is the features vector of the training example *m*, $a^{m}$ is the class label of the training example *m*, and m∈{1,2,…,M}. Each class $a^{m}$ incorporates the optimal combination of the MCS and the payload length for the corresponding features vector $f^{m}$. The features of the training example *m* can be extracted from the corresponding estimated OFDM channel coefficients, ${\hat{H}}_{p}^{m}\left[n\right]$, at the all $U_{s}$ used subcarriers and the estimated noise standard deviation, ${\hat{\sigma }}^{m}$. It can be represented by

(9) $$f^{m}=\left[\left|{\hat{H}}_{p}^{m}\left[1\right]\right|,\left|{\hat{H}}_{p}^{m}\left[2\right]\right|,\ldots ,\left|{\hat{H}}_{p}^{m}\left[U_{s}\right]\right|,{\hat{\sigma }}^{m}\right]$$

where $\left|{\hat{H}}_{p}^{m}\left[n\right]\right|$ is the magnitude of the estimated channel coefficient ${\hat{H}}_{p}^{m}\left[n\right]$. The key performance metrics that we use to classify the training examples are the effective throughput, Γ, and the FER. The class label $a^{m}$ of training example *m* is the optimal combination of the MCS and the payload length that achieves the maximum throughput and complies with a predefined FER constraint, ϵ, as expressed by the following equation

(10) $$a^{m}=\arg\max_{a}\left\{\Gamma \left(a,f^{m}\right):FER\left(a,f^{m}\right)<\epsilon \right\},$$

where $\Gamma (a,f^{m})$ is the throughput achieved at the channel condition corresponding to the features vector $f^{m}$ with the transmission class *a*, and $FER(a,f^{m})$ is the FER at the channel condition corresponding to the features vector $f^{m}$ with the transmission class *a*. Note that (10) is similar to (8), except that the class $a^{m}$ replaces $\left\{i^{*},l^{*}\right\}$ and the features vector $f^{m}$ is used instead of the intractable actual channel condition. With (9) and (10), we can generate many training examples ${\left\{f^{m},a^{m}\right\}}_{m=1}^{M}$ to train the DCNN for AMCPLS. (The generation of the training set is described in detail in Section 5.2).

### 4.2. Deep Convolutional Neural Network Structure

**Figure 3:**
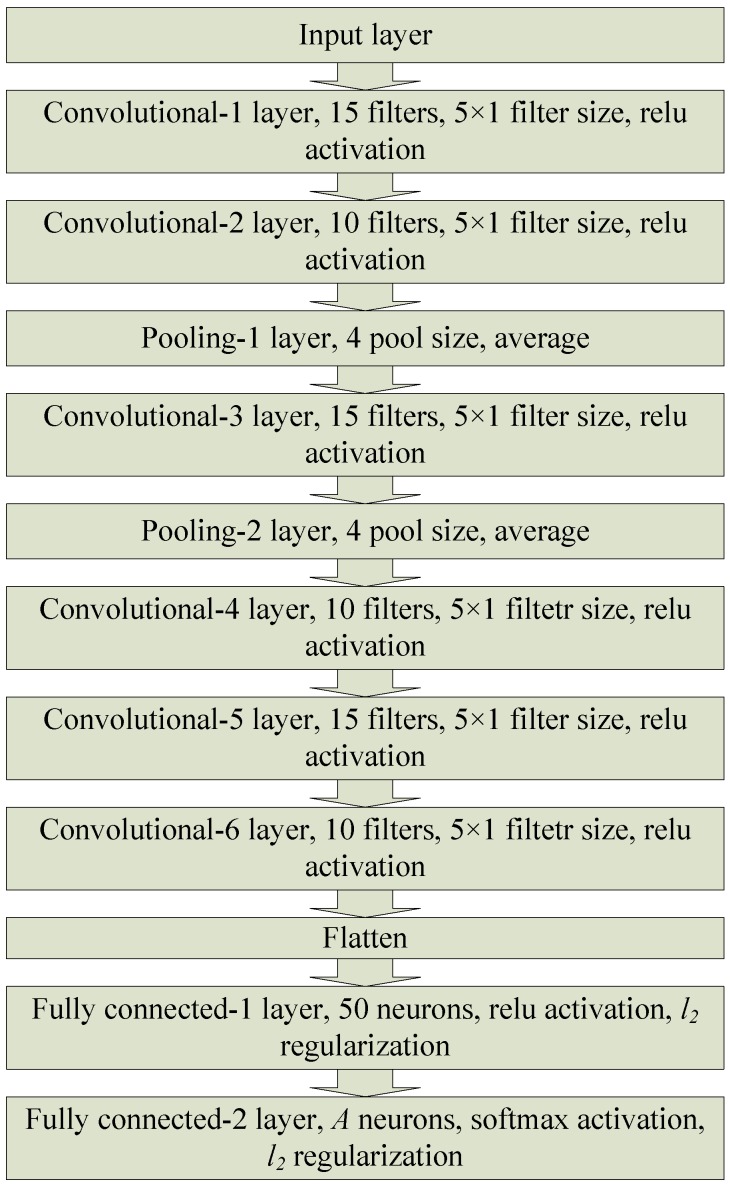
The proposed deep convolutional neural network model.

The structure of the used deep convolutional neural network (DCNN) incorporates six convolutional layers, two average pooling layers, and two fully connected layers, as depicted in Figure 3 and Algorithm 1. Particularly, each of the first, third and fifth convolutional layers has 15 filters. Also, each of the second, fourth, and sixth convolutional layers has 10 filters. All convolutional layers utilize filters of size 5×1 and rectified linear unit (ReLU) activation function. One of the average pooling layers is after the second convolutional layer, and the second average pooling layer is after the third convolutional layer. Each of the pooling layers has a pool size of four. The first fully connected layer has 50 neurons with ReLU activations. The last layer is a fully connected layer with a number of neurons that is equal to the number of classes. This last layer utilizes softmax activation function. The second norm regularization is adopted in the two fully connected layers in order to alleviate the effect of overfitting [22]. The adam optimizer [25] along with the categorical cross entropy loss function are used for training the DCNN. The DCNN is trained for 100 epochs with a batch size of 100.

After the DCNN is trained, the receiver can employ it to anticipate the appropriate transmission class, i.e., the optimal combination of MCS and payload length. Then, the receiver feedbacks the MCS and payload length to the transmitter for considering them in the transmission of the next frame. Note that although the channel is varying over different frames, we use the channel estimates of the current frame to construct the input features to DCNN for predicting the suitable transmission class of the next frame. Since the channels between two adjacent frames have high correlations, this feedback mechanism is still rather robust (please see the performance evaluation of our DCNN based AMCPLS scheme in Section 5.2).

**Algorithm 1** Proposed DCNN structure
  1:Input layer  2:First convolutional layer with 15 filters, each of size 5 × 1, and relu activation.  3:Second convolutional layer with 10 filters, each of size 5 × 1, and relu activation.  4:First average pooling layer with pool size of 4.  5:Third convolutional layer with 15 filters, each of size 5 × 1, and relu activation.  6:Second average pooling layer with pool size of 4.  7:Fourth convolutional layer with 10 filters, each of size 5 × 1, and relu activation.  8:Fifth convolutional layer with 15 filters, each of size 5 × 1, and relu activation.  9:Sixth convolutional layer with 10 filters, each of size 5 × 1, and relu activation. 10:Flatten layer. 11:First fully connected layer with 50 neurons, relu activation, and $l_{2}$ regularization. 12:Second fully connected layer with *A* neurons, softmax activation, and $l_{2}$ regularization.


We remark here that although the training of the DCNN for AMCPLS needs extensive computational resources and is time-consuming, it can be implemented offline in clouds. After the DCNN weights are finalized by the training process, we deploy the trained DCNN in the receiver of vehicles for making online AMCPLS. The implementation of DCNN on vehicels can employ FPGA or ASIC that can be optimized to compute the outputs of neurons in parallel. Furthermore, unlike the training process, the prediction process of DCNN does not require heavy processing as it involves only matrix multiplication, addition and nonlinear activation. Therefore, the proposed DCNN for AMCPLS can actually have a lower computation latency.

## 5. Numerical Results

In this section, we utilize simulations to evaluate the proposed AMCPLS scheme for the IEEE 802.11p V2V communications. The simulation parameters of the considered IEEE 802.11p system are shown in Table 2. We consider channel equalizations that are based on both traditional LS channel estimation [14] and STA channel tracking [13]. Furthermore, we compare the performance of our proposed DCNN based AMCPLS with the performance of other rival machine learning algorithms such as k-NN and SVM. In our evaluations we assume that the set of allowable payload lengths is L={100,300,500} Bytes. Furthermore, for the IEEE 802.11p, the set of available MCSs is I={0,1,2,3,4,5,6,7}. Thus the set of all possible classes is A={0,1,2,…,23}. In our simulations, we assume that the target FER, ϵ is 0.05. Furthermore, we assume a rural LOS environment [9,26]. In our evaluations, we utilize both the effective throughput and the FER. We have used the FER rather than the bit error rate (BER) because our target is to select both the MCS and the payload length to maximize the effective throughput under FER constraint and the BER is independent on the payload length.

### 5.1. Performance of Ideal AMCPLS

Practically, the ideal AMCPLS expressed in (8) can not be implemented as we do not know the actual channel condition and the FER. However, we can evaluate it using numerical enumerations, i.e., we try all possible combinations of the MCSs and payload lengths at every SNR value; then, we select the combination of the MCS and payload length that achieves the maximum effective throughput and satisfies the predefined FER. In this regard, we evaluate the performance of ideal AMCPLS, where we assume that both the actual channel and the FER can be obtained beforehand (the FER is obtained from simulations using the actual channel) and thus the ideal combination of the MCS and the payload length can be determined for the transmission of the current frame.

**Figure 4:**
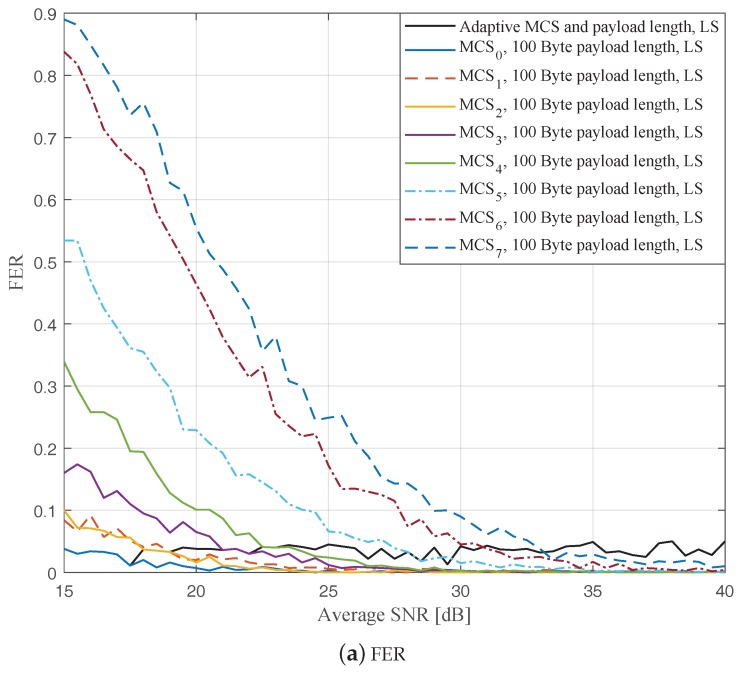

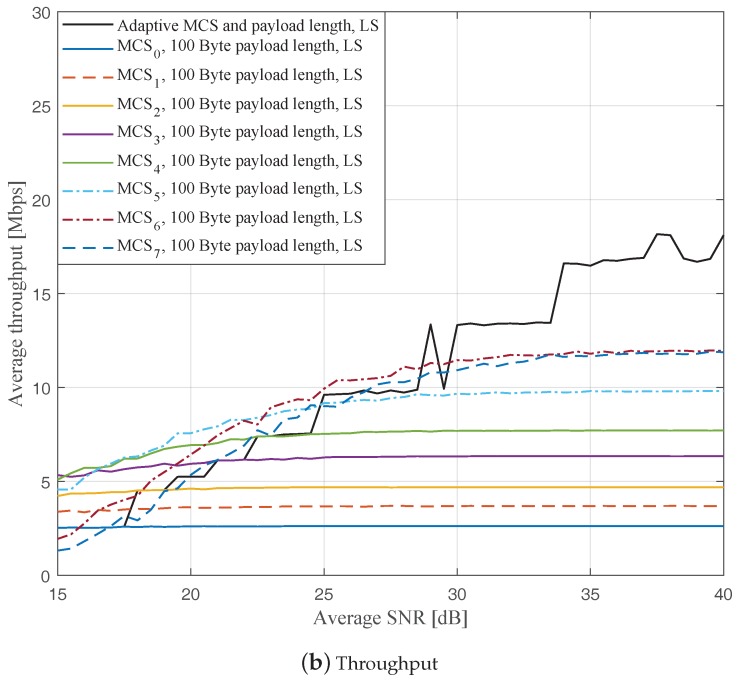
Comparison among the proposed AMCPLS and the fixed 100 Bytes payload length at different MCSs when traditional LS channel estimation is used in the rural LOS V2V environment.

**Figure 5:**
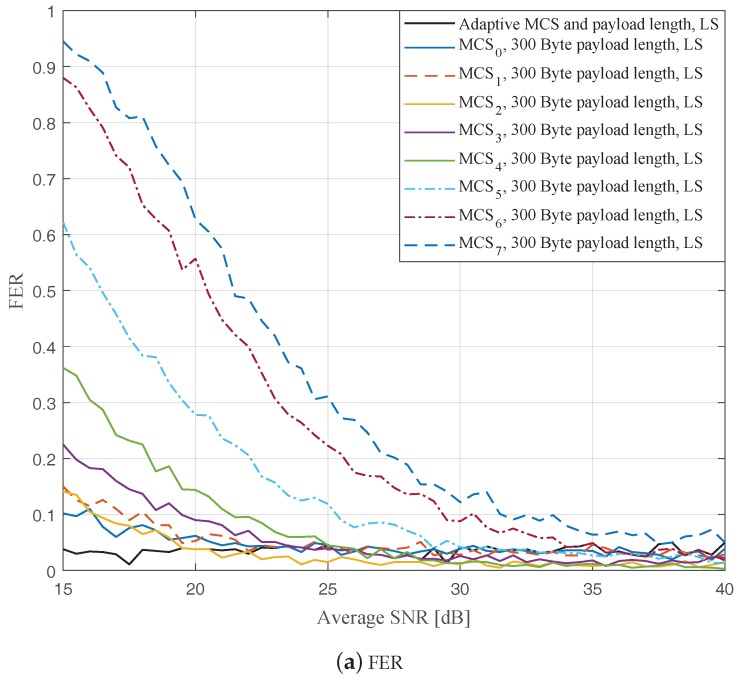

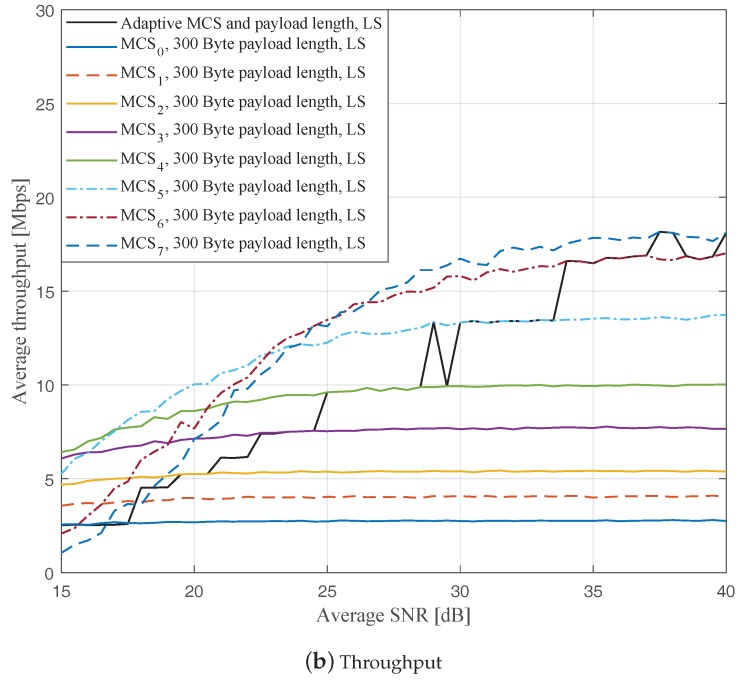
Comparison among the proposed AMCPLS and the fixed 300 Bytes payload length at different MCSs when traditional LS channel estimation is used in the rural LOS V2V environment.

**Figure 6:**
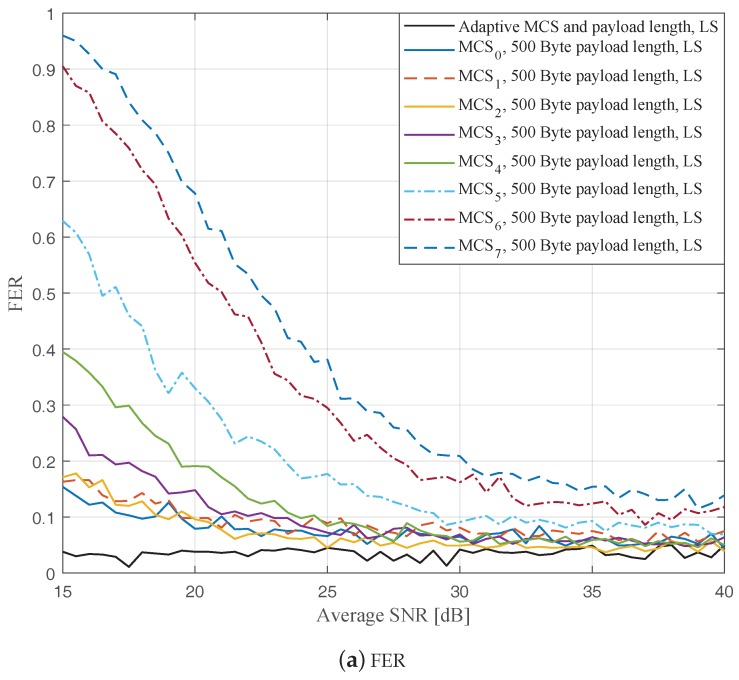

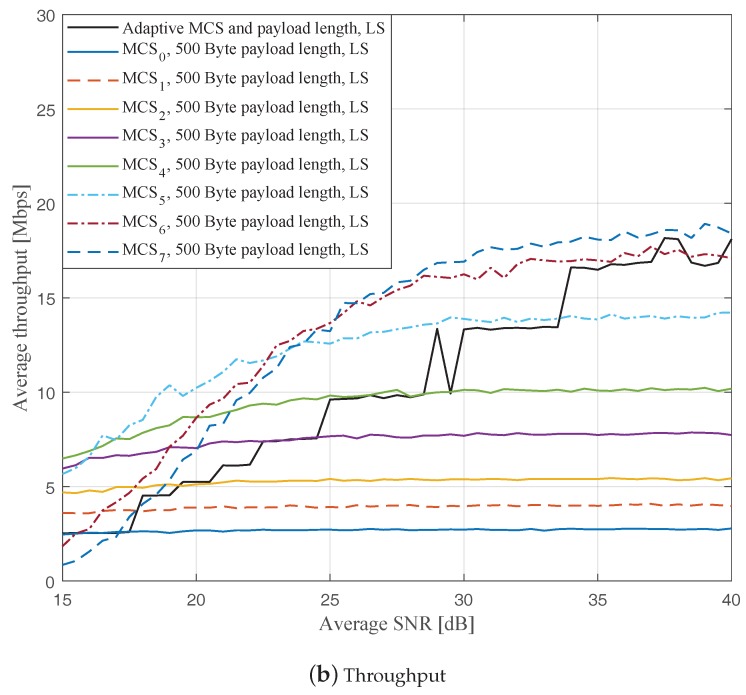
Comparison among the proposed AMCPLS and the fixed 500 Bytes payload length at different MCSs when traditional LS channel estimation is used in the rural LOS V2V environment.

**Figure 7:**
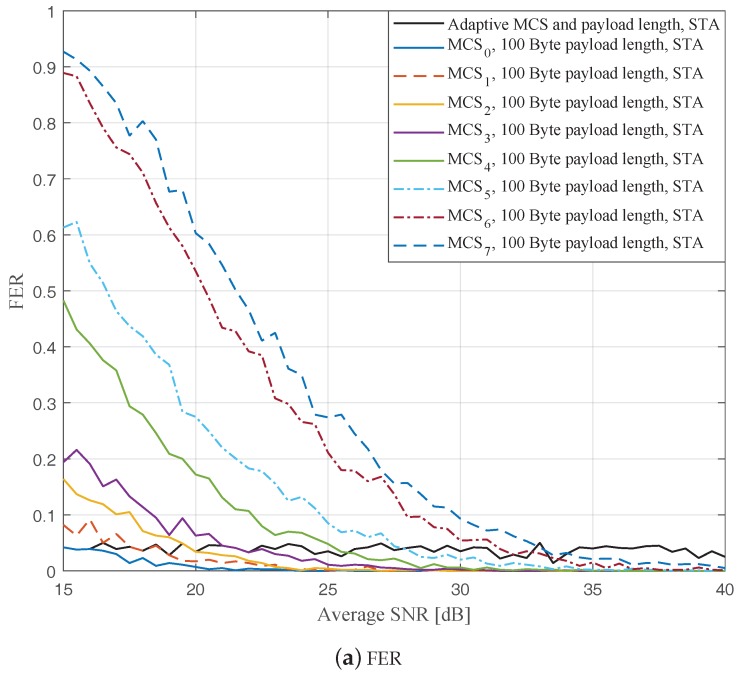

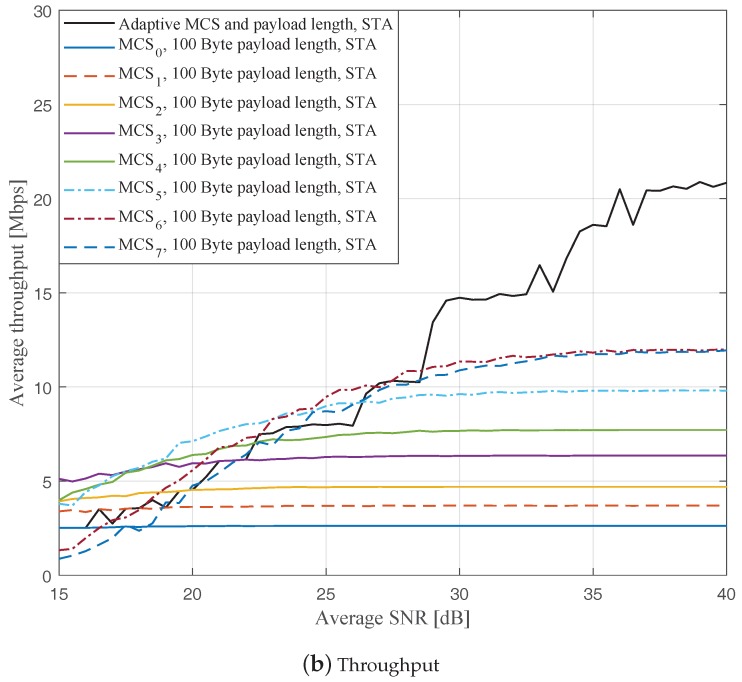
Comparison among the proposed AMCPLS and the fixed 100 Bytes payload length at different MCSs when STA channel tracking is used in the rural LOS V2V environment.

**Figure 8:**
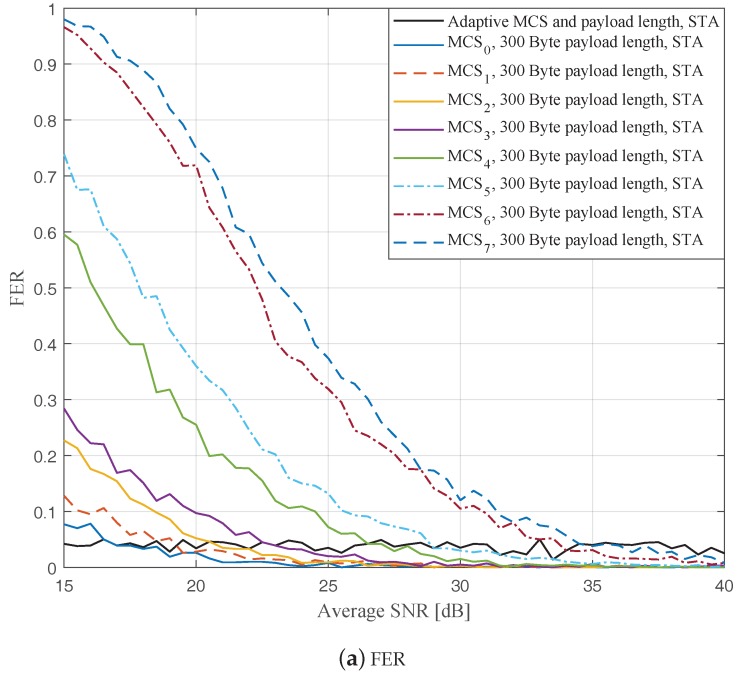

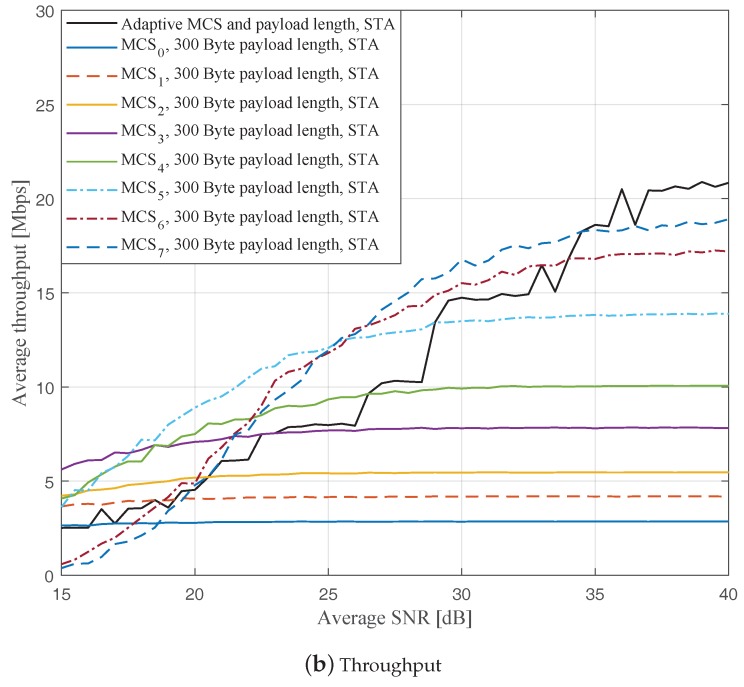
Comparison among the proposed AMCPLS and the fixed 300 Bytes payload length at different MCSs when STA channel tracking is used in the rural LOS V2V environment.

**Figure 9:**
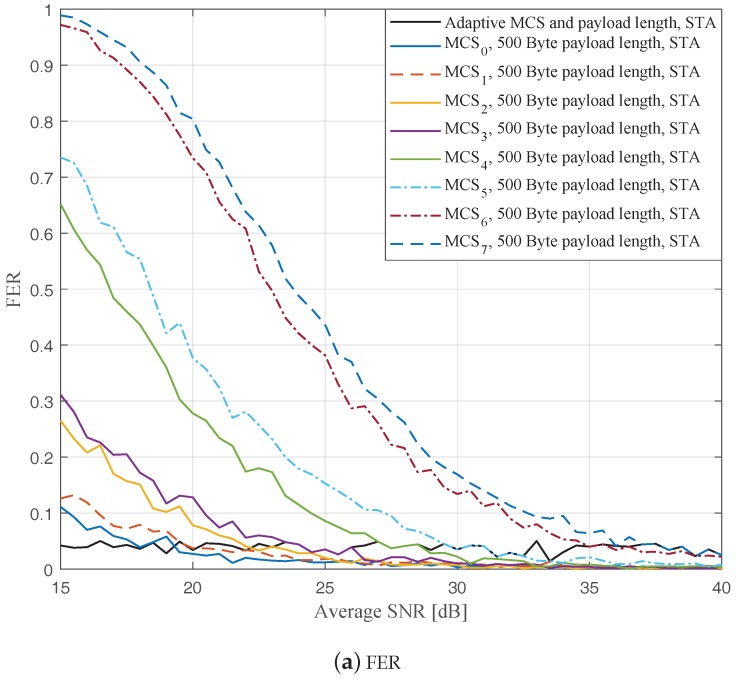

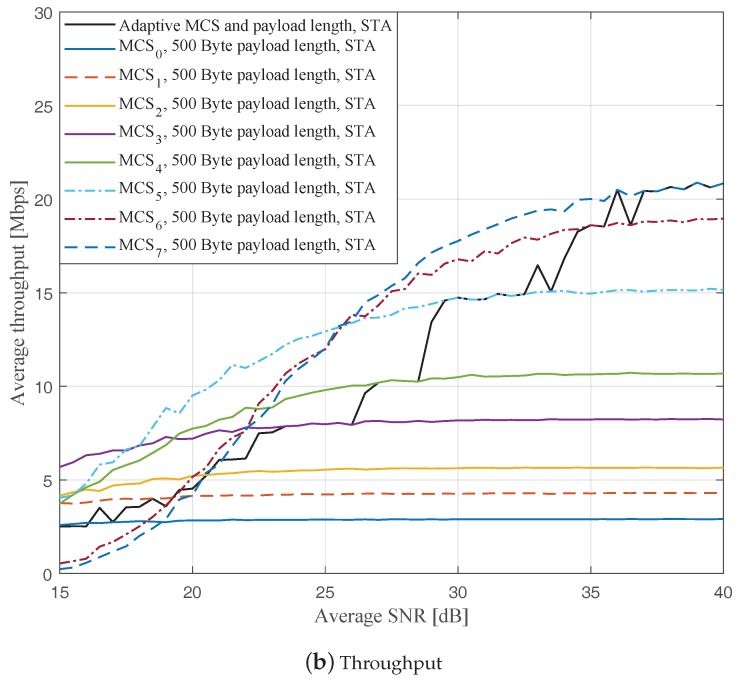
Comparison among the proposed AMCPLS and the fixed 500 Bytes payload length at different MCSs when STA channel tracking is used in the rural LOS V2V environment.

In this part, we perform simulations to evaluate the ideal AMCPLS scheme considering both the traditional LS channel estimation [14] and the STA channel tracking [13]. We consider the V2V communication in the rural LOS environment at different signal to noise ratios (SNR) that range from 15 dB to 40 dB. Specifically, Figure 4, Figure 5 and Figure 6 depict the FERs and effective throughputs of the proposed AMCPLS scheme compared to fixed MCSs with fixed payload lengths of 100, 300, and 500 Bytes, respectively, when the traditional LS channel estimation is employed. It can be observed from the results that for most of the fixed schemes, the FER decreases and the throughput increases as the SNR increases. Furthermore, increasing the payload length when a specific MCS is utilized leads to an increase in the effective throughput due to the overhead reduction; at the same time, the FERs for most of the fixed MCSs increase as well, which impact the reliability and energy efficiency of the V2V communication link. On the other hand, the proposed AMCPLS scheme always satisfies the predefined target FER, ϵ=0.05 and provides satisfactory throughputs. Similarly, Figure 7, Figure 8 and Figure 9 show the FERs and effective throughputs of the proposed AMCPLS scheme compared to fixed MCSs with fixed payload lengths of 100, 300, and 500 Bytes, respectively, when the STA channel tracking is utilized. It is noteworthy that, for fixed MCS with fixed payload length schemes, the improvements in the FERs and throughputs of the STA channel tracking compared to that of the traditional LS channel estimation appears significantly at high SNR values especially when a long payload length is used. For the AMCPLS, the FER constraint is always satisfied for both the STA channel tracking and the traditional LS channel estimation. The AMCPLS when combined with the STA channel tracking can provide significant improvement in the effective physical layer throughput when compared to traditional LS channel estimation, specifically at high SNR values.

It is noteworthy that the effective throughput of the ideal AMCPLS scheme sometimes fluctuates even for an increase in the average SNR value when both LS channel estimation and STA channel tracking are considered as depicted in Figure 4b, Figure 5b, Figure 6b, Figure 7b, Figure 8b and Figure 9b. These fluctuations are for small SNR variations where the fading effect has the dominant impact on the channel condition. At these fluctuation points, the fading effect may make the channel condition accompanied with the low average SNR value better than the channel condition accompanied with the high average SNR value because the difference in the SNR is small (about 0.5 dB).

### 5.2. Performance Evaluation of Machine Learning Based AMCPLS

In this part, we evaluate the proposed DCNN based AMCPLS approach. The k-NN based AMCPLS and the SVM based AMCPLS are also evaluated and their performance are used as benchmarks. We perform simulations for IEEE 802.11p V2V communication systems in the rural LOS environment. The training examples ${\left\{f^{m},a^{m}\right\}}_{m=1}^{M}$ are generated as follows. We simulate the IEEE 802.11p V2V system at different SNR values that range from 15 dB to 40 dB with a 1 dB step. For every SNR value, transmission using all possible classes (indexed from 0 to 23) is performed for different 1000 channel realizations and the FER of each class is calculated. Then, the ideal class, that corresponds to the maximum throughput and at the same time satisfies the target FER, ϵ=0.05, is selected as the label for the channel realizations of the corresponding SNR. For every frame received correctly, the features $f^{m}$ are computed and saved along with the corresponding label $a^{m}$. The feature set extracted as in (9) is used to train the DCNN, k-NN, and SVM for AMCPLS.

After we have finished the training, in order to evaluate the proposed DCNN AMCPLS approach, we generate new 1000 channel realizations for each SNR value that ranges from 15 dB to 40 dB with a 0.5 dB step. Transmissions are performed over all channel realizations. At the receiver, the features are computed from the received signal. Then, the features are used as the input to the trained DCNN, k-NN, and SVM for predicting the corresponding class, i.e., optimal combination of MCS and payload length that should be utilized in the next frame.

**Figure 10:**
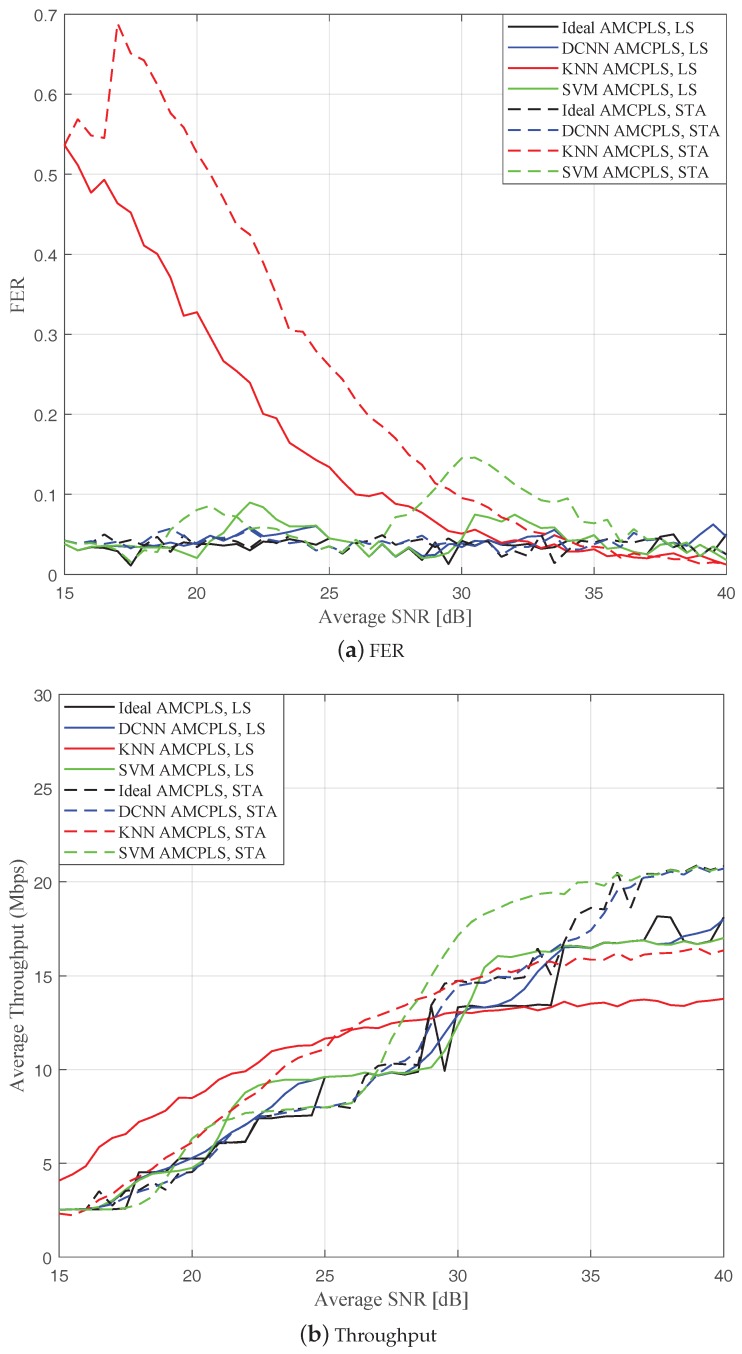
Comparison among the proposed DCNN AMCPLS, k-NN AMCPLS, and SVM AMCPLS for both traditional LS channel estimation and STA channel tracking in the rural LOS V2V environment.

Figure 10 depicts the FER and effective throughput performance of the proposed DCNN AMCPLS, k-NN AMCPLS, and SVM AMCPLS when both traditional LS channel estimation and STA channel tracking are used. Particularly, Figure 10a shows that k-NN AMCPLS and SVM AMCPLS failed to fulfill the target FER constraint for a considerable range of SNR values when both traditional LS channel estimation and STA channel tracking are considered. On the other hand, the proposed DCNN AMCPLS approach has better FER performance compared to both k-NN AMCPLS and SVM AMCPLS for both the traditional LS channel estimation and STA channel tracking. From Figure 10b, it is noted that the k-NN AMCPLS and SVM AMCPLS can provide higher throughput than the proposed DCNN AMCPLS at some ranges of SNR values for both traditional LS channel estimation and STA channel tracking at the price of the increased FER. The increased FERs of both k-NN AMCPLS and SVM AMCPLS affect their reliability in V2V communications and requires multiple frame retransmissions that affects the system latency and energy efficiency.

In order to assess the computational cost of the DCNN AMCPLS, the k-NN AMCPLS, and the SVM AMCPLS, we evaluated the average time required by each approach for predicting the transmission mode class of more than 49,000 channel realizations on an Intel core i3-6100 with speed 3.7 GHz processor. We found that the average prediction times of the DCNN AMCPLS, k-NN AMCPLS, and SVM AMCPLS are 64 µs, 480 µs, and 1194 µs, respectively. It is clear that the proposed DCNN AMCPLS has reduced prediction time and reduced FER compared to both k-NN AMCPLS and SVM AMCPLS. This means the proposed DCNN AMCPLS is more reliable than both k-NN AMCPLS and SVM AMCPLS for safety related related applications, where the increased FER and the increased transmission mode class prediction time may lead to severe consequences such as cars collisions.

## 6. Conclusions

This work proposes an adaptive scheme for selecting both the MCS and payload length in IEEE 802.11p V2V communication systems. We consider practical receivers that involve realistic timing synchronization, carrier frequency offset correction and channel estimation. Furthermore, we employ a deep learning approach to solve the AMCPLS scheme. We design a deep convolutional neural network in order to be trained using the estimated channel coefficients and the noise standard deviation as input features. Our deep learning approach can predict the suitable combination of the payload length along with the modulation and coding scheme. Simulation results show that the proposed AMCPLS scheme outperforms fixed MCS and payload length schemes. In addition, the proposed DCNN AMCPLS approach has better prediction time, frame error rate, and effective physical layer throughput performance compared to both k-NN AMCPLS, and SVM AMCPLS approaches. In a future work, we will consider the application of the proposed AMCPLS scheme in the broadcasting scenario.

## Figures and Tables

**Table 1:** IEEE 802.11P modulation and coding schemes [13].

MCS	Data Rate [Mbps]	Modulation	Channel Coding Rate	Coded Bits per Subcarrier $\left(N_{\mathrm{BSC}}\right)$	Data Bits per OFDM Symbol $\left(N_{\mathrm{DBOS}}\right)$
0	3	BPSK	1/2	1	24
1	4.5	BPSK	3/4	1	36
2	6	QPSK	1/2	2	48
3	9	QPSK	3/4	2	72
4	12	16-QAM	1/2	4	96
5	18	16-QAM	3/4	4	144
6	23	64-QAM	2/3	6	192
7	27	64-QAM	3/4	6	216

**Table 2:** Simulation parameters.

Carrier frequency	5.9 GHz
Channel bandwidth	10 MHz
OFDM symbol duration	8 µs
IFFT period	6.4 µs
Gaurd interval	1.6 µs
The total number of subcarriers	64
The number of data subcarriers	48
The number of pilot subcarriers	4
The number of null carriers	12
The number of short training symbols	10
The duration of a short training symbol	1.6 µs
The number of long training symbols	2
The duration of a long training symbol	6.4 µs
Target FER	0.05

## Abbreviations

The following abbreviations are used in this manuscript:

ITS	Intelligent Transportation Systems
V2V	vehicle-to-vehicle
AMCPLS	Adaptive Modulation, Coding and Payload Length Selection
FER	Frame Error Rate
DCNN	Deep Convolutional Neural Network
k-NN	k-Nearest Neighbors
SVM	Support Vector Machines
DSRC	Dedicated Short-Range Communication
AMC	Adaptive Modulation and Coding
WLAN	Wireless Local Area Network
MCS	Modulation and Coding Scheme
MIMO	Multiple-Input, Multiple-Output
OFDM	Orthogonal Frequency Division Multiplexing
LS	Least Squares
STA	Spectral Temporal Averaging
BPSK	Binary Phase Shift Keying
QPSK	Quadrature Phase Shift Keying
QAM	Quadrature Amplitude Modulation
5G	Fifth Generation
4G	Fourth Generation
LTE	Long-Term Evolution
CSI	Channel State Information
IFFT	Inverse Fast Fourier Transform
STS	Short Training Symbol
LTS	Long Training Symbol
CP	Cyclic Prefix
AWGN	Additive White Gaussian Noise
ReLU	Rectified Linear Unit
FPGA	Field-Programmable Gate Array
ASIC	Application-Specific Integrated Circuit
BER	Bit Error Rate
SNR	Signal to Noise Ratio
LOS	Line of Sight
